# Anti-oppression pedagogy in health professions: a scoping review

**DOI:** 10.1007/s10459-024-10336-0

**Published:** 2024-05-13

**Authors:** Meredith Smith, Tricia McGuire-Adams, Kaylee Eady

**Affiliations:** 1 Department of Physical Therapy, University of Toronto, Toronto, ON Canada; 2 Faculty of Kinesiology and Physical Education, University of Toronto, Toronto, ON Canada; 3 Faculty of Education, University of Ottawa, Ottawa, ON Canada

**Keywords:** Antiracism, Decoloniality, Health inequity, Structures of oppression, Systems of oppression, Health professions education

## Abstract

Health professional learners are increasingly called to learn about health inequity to reduce inequities and improve patient care and health outcomes. Anti-oppression pedagogy (AOP) addresses the need for health professional learners to understand multiple health inequities and the structures and systems that produce inequities. However, the inclusion of AOP in health professions education varies and there is a lack of clarity in its conceptualization and integration. A scoping review was conducted to address this gap and to understand how AOP is conceptualized and integrated in health professions education. Thirty-six articles met the inclusion criteria. The articles demonstrated that AOP is not commonly utilized terminology within health professions education. When AOP is integrated, it is not consistently conceptualized but is generally viewed as a broad concept that focuses on antiracism; decoloniality; intersectionality; and supporting learners to understand, critically reflect on, and act against structural and systemic forms of oppressions. In addition, there is variation in the integration of AOP in health professions education with the most common methods consisting of discussions, cases, reflection, learning through lived experiences, and the incorporation of humanities within a longitudinal curriculum. The results of this scoping review highlight the need for health professions education to develop one clear concept that educators use when teaching about anti-oppression, which may reduce working in silos and allow educators to better collaborate with each other in advancing this work. In addition, this review suggests that health professional programs should consider incorporating AOP in curricula with a broad and longitudinal approach utilizing the common methods of delivery. To better support programs in including AOP in curricula, further research is required to emphasize the benefits, provide clarity on its conceptualization, and determine the most effective methods of integration.

## Introduction

There are increasing calls for health professional learners to learn about health inequity (Maldonado et al., 2014; Rosa et al., 2021; Sharma & Kuper, 2017) and a rising focus on the importance of education on the systems and structures that result in health inequity (Bowleg, 2012; Drevdahl, 2018; Metzl & Petty, 2017). This aligns with growing literature correlating health inequity and unfavorable social determinants of health (SDoH) with poor health outcomes and increased morbidity and mortality (Bundy et al., 2023; Chen & Krieger, 2021; L’Hôte et al., 2022; Metzl et al., 2020). The World Health Organization defines SDoH as, the “conditions in which people are born, grow, work, live, and age, and the wider set of forces and systems shaping the conditions of daily life” (World Health Organization, n.d.). SDoH impact health outcomes and influence health inequities which are the “unfair and avoidable differences in health status seen within and between countries” (World Health Organization, n.d.). Structures (e.g., economic conditions) and systems (e.g., racism, heterosexism, sexism, classism) of oppression produce health inequities (Bowleg, 2012; Metzl & Hansen, 2014; Metzl & Petty, 2017) and intersect to maintain disparities (Bowleg, 2012).

The expectations for learners to understand health inequities is increasingly embedded in many health professional programs. For example, in medicine, recent diversity, equity, and inclusion competencies state that medical school graduates should be able to identify “systems of power, privilege, and oppression and their impacts on health outcomes” (Association of American Medical Colleges, 2022, p. 9). In nursing, guidelines state that education and training should “promote mandatory health equity education in all levels of nursing curricula” and “teach students to critically apply health equity concepts” (Rosa et al., 2021, p. 17). In social work, one of the competencies states that social workers should “understand the societal and historical roots of social and racial injustices and the forms and mechanisms of oppression and discrimination” (Council on Social Work Education, 2022, p. 9).

However, teaching about health inequities in health professions often suggests that health inequities are due to cultural or ethnic issues which can reinforce stereotypes and avoids looking at the drivers of inequity (Blanchet Garneau et al., 2021; Chin, 2021). For example, health inequity content that focuses on Indigenous cultures instead of colonial systems may reinforce cultural stereotypes and othering, which further oppress Indigenous communities (Blanchet Garneau et al., 2021), and avoids discussions and learning about colonialism. Teaching about structures and systems of oppression helps learners to understand the root causes of health inequities, consider factors beyond the individual which impact health, and address health issues (Bowleg, 2012; Metzl & Hansen, 2014). Therefore, educators are encouraged to include content, within curricula, on the impact of structural factors on health (Bowleg, 2012; Drevdahl, 2018; Metzl & Petty, 2017). Anti-oppression pedagogy (AOP) achieves this goal.

### Reflexive statement

Positionality is important to situate the authors’ identities and experiences, which informs part of the rationale for the work. We each bring lived and academic experiences to the multifaced and complex engagement with anti-oppression and identity. The first author is a Black, able-bodied, cisgender female. She is a physiotherapist and an Assistant Professor in the Teaching Stream within the Physical Therapy Department at the University of Toronto. She is committed to work that advances equity and anti-oppression practice in healthcare and health professions education.

The second author is an Anishinaabe woman from Bingwi Neyaashi Anishinaabek and is an Associate Professor with the Faculty of Kinesiology and Physical Education at the University of Toronto. Through her scholarship and teaching, she seeks to disrupt ongoing settler colonialism by centring Indigenous ways of being in physical activity, health, and wellbeing to foster Indigenous resurgence.

The third author is a White, able-bodied, cisgender woman, and mother of two biracial children. She is an Assistant Professor of Health Professions Education in the Faculty of Education at the University of Ottawa. Through her academic activities and patient/community partnerships, she aims to improve the teaching and learning of health professionals and with this, the care provided to and the health of our communities.

### Anti-oppression pedagogy

AOP has been defined in multiple ways and generally refers to teaching about oppression, the systems that contribute to oppression, and the disruption of oppression (Aqil et al., 2021; Galloway et al., 2019; Lavallée, 2014; Lyons et al., 2023). It facilitates understanding how systems of oppression influence inequity (Aqil et al., 2021; Lyons et al., 2023) and it challenges systems and structures that create and maintain disparities. AOP addresses a lack of knowledge and a resistance to knowledge that demonstrates one’s complicity with structures and systems of oppression. Therefore, in addition to learning, AOP requires one to unlearn or relearn what was previously known about oppression and space is required for learners to work through this process (Galloway et al., 2019; Kumashiro, 2000). AOP also involves self-reflexivity which requires learners to not only consider their involvement in the dynamics of oppression but also to use this knowledge to rethink their own sense of self (Kumashiro, 2000). A critical lens is often incorporated in anti-oppression work to critique and question systems of oppression and one’s own assumptions and to recognize the “masking of privilege” (Kumashiro, 2000, p. 37; Lavallée, 2014).

In addition, anti-oppression considers multiple forms of oppression and the intersection between different systems of oppression such as gender, sexual orientation, race, ability, and colonization (Lavallée, 2014). “Intersectionality recognizes that one form of oppression does not exist in isolation” (Lavallée, 2014, p. 2) and is a component of AOP. AOP has also been defined as tackling problems of power and access to resources (Harlow & Hearn, 1996); recognizing power imbalances and promoting change to redress the balance of power (Dalrymple & Burke, 1998, p. 25); engaging in discussions of privilege, racism, and the systems that sustain them (Galloway et al., 2019); and teaching about decolonization which is concerned with dismantling the oppression of Indigenous peoples and oppression from colonization (Jakubec & Bourque Bearskin, 2020).

AOP addresses the need for health professional learners to understand multiple health inequities and the structures and systems that produce inequities. Yet, the inclusion of AOP in health professions education varies. Medical education does not typically include content on understanding the drivers of inequities (Punchhi et al., 2022) and there has been minimal focus on anti-oppression practice in nursing (Ng & Wai, 2021). Social work focuses more on anti-oppressive practice and the drivers of inequities (Ng & Wai, 2021; Punchhi et al., 2022). As such, there is a lack of clarity in how AOP is conceptualized and integrated across health professions education.

## Methods

This scoping review was conducted using Arksey and O’Malley’s (2005) five-step framework and informed by recommendations from Levac et al. (2010), Peters et al. (2020), and Pollock et al. (2023). The Preferred Reporting Items for Systematic reviews and Meta-Analyses extension for Scoping Reviews (PRISMA-ScR; Tricco et al., 2018) guided the reporting of this study. The five-step framework for this project included the following:

### Stage 1: identifying the research question

The first step of a scoping review is identifying the research question (Arksey & O’Malley, 2005) with recommendations for a clear scope of inquiry (Levac et al., 2010) that incorporates the ‘‘PCC’’ (i.e., population, concept, and context) mnemonic (Peters et al., 2020). As previously situated, the purpose of this review was to explore AOP in health professions education. The research question was: How is AOP conceptualized and integrated in health professions education curricula?

To clarify the scope of inquiry, health professions education was defined as education for regulated health professional learners. AOP was clarified as aiming to teach learners about the systems and root causes of oppression (Aqil et al., 2021; Lyons et al., 2023).

### Stage 2: identifying relevant studies

A search strategy was developed in consultation with a research librarian. The search was implemented in the following three databases: Medline, Embase, and CINAHL. These databases were chosen based on the focus on the medical field, which includes health professions. The search strategy focused on terms related to anti-oppression, anti-racism, and decolonization due to the scope of this project and because of the increased emphasis on anti-oppression following the Truth and Reconciliation Commission of Canada: Calls to Action (2015), the police killings of George Floyd and others, and the racial disparities observed in the coronavirus disease 2019 pandemic (Churchwell et al., 2020). The search strategy included the following keywords: “antioppress*” or “anti oppress*” OR “decolon*” “antiraci*” or “anti raci*” AND “training” or “education” or “instruction” or “course*” or “workshop*” or “curricul*” or “pedagog*” AND “learner*” or “student*” or “trainee*” or “mentee*” or “residen*”.

The type of literature was not restricted, and the dates were restricted from January 2015 to March 2023. The dates were selected due to the increased focus on anti-oppression following the Truth and Reconciliation Commission of Canada: Calls to Action (2015) and the formation of the first Canadian chapter of Black Lives Matter Canada in 2014 (Black Lives Matter Canada, 2022). The search was completed on March 3, 2023. References were imported into Covidence, a software tool to manage components of systematic and scoping reviews. Duplications were identified and removed by Covidence.

### Stage 3: study selection

The first and second authors independently reviewed all titles and abstracts in Covidence. Disagreements were resolved via discussion and eligibility was refined and clarified during this discussion. Similarly, full articles were independently reviewed by these two authors and disagreements were resolved via discussion.

#### Eligibility

Articles were included in the study if (a) AOP or teaching learners about anti-oppression was a key concept, (b) AOP was for a health professional program, (c) the learners were in a regulated health professional program, and d) the article was written in English.

AOP was defined as education that was “concerned with recognizing, acknowledging and taking action against oppression” (Lavallée, 2014, p. 40), addressing all forms and multiple systems of oppression, recognizing the ways multiple forms of oppression intersect (Lavallée, 2014), and focusing on understanding the root causes of oppression (Aqil et al., 2021; Lyons et al., 2023). During discussions about the screening of abstracts and titles, the authors decided that more clarity was needed in the meaning of AOP. At this point, the term was refined to include any of the following: “concerned with recognizing, acknowledging and taking action against oppression” (Lavallée, 2014, p. 40); addresses all forms and multiple systems of oppression or recognizes the ways the multiple forms of oppression intersect (Lavallée, 2014); focuses on teaching about the root causes of oppression (Aqil et al., 2021; Lyons et al., 2023); tackles problems of power and access to resources (Harlow & Hearn, 1996); recognizes power imbalances and promoting change to redress the balance of power (Dalrymple & Burke, 1998, p. 25); engages in discussions of privilege and racism and the systems that sustain them (Galloway et al., 2019); teaches about decolonization which is specifically concerned with dismantling the systematic oppression of Indigenous peoples, Indigenous culture, and oppression from colonization (Jakubec & Bourque Bearskin, 2020); or uses a critical lens to educate about anti-oppression, racism, power, and privilege (Kumashiro, 2000; Lavallée, 2014).

Articles were excluded for not meeting the inclusion criteria. During discussions about the screening of abstracts and titles, the exclusion criteria was further refined and articles were excluded if (a) the education consisted of a single workshop or session, (b) learners were not a focus of the education, (c) the participant’s perspective was provided without information on the education; (d) the focus was on how to teach in an anti-oppressive manner versus teaching about anti-oppression; and (e) the text was not an article (e.g., an abstract only). There were 244 articles excluded.

### Stage 4: charting the data

A data charting form was developed focusing on the way AOP was conceptualized and integrated. As recommended by Pollock et al. (2023), only items related to the scoping review questions were extracted. The data charting form was refined after extracting data from five articles. The revised data charting form was reviewed by two of the authors, and the form was finalized through discussion. The first author extracted the following data from each article: author(s); year; country; purpose; study design; type of learners; main theories and concepts on which the education was based; conceptualization of AOP; description of the curriculum; and methods of integrating content in the curriculum. See Appendix for a summary of the information collected in the data charting form.

### Stage 5: collating, summarizing, and reporting the results

Collating of the data was guided by exploring the manner in which AOP was conceptualized and integrated in health professions education. Based on the recommendations of Peters et al. (2020) and Pollock et al. (2023), a basic descriptive analysis was completed. The findings captured were the general study characteristics, the focus of the curriculum, the conceptualization of AOP, the extent to which the concepts aligned or overlapped with AOP if AOP was not conceptualized, and the methods for integrating content.

## Results

**Fig. 1:**
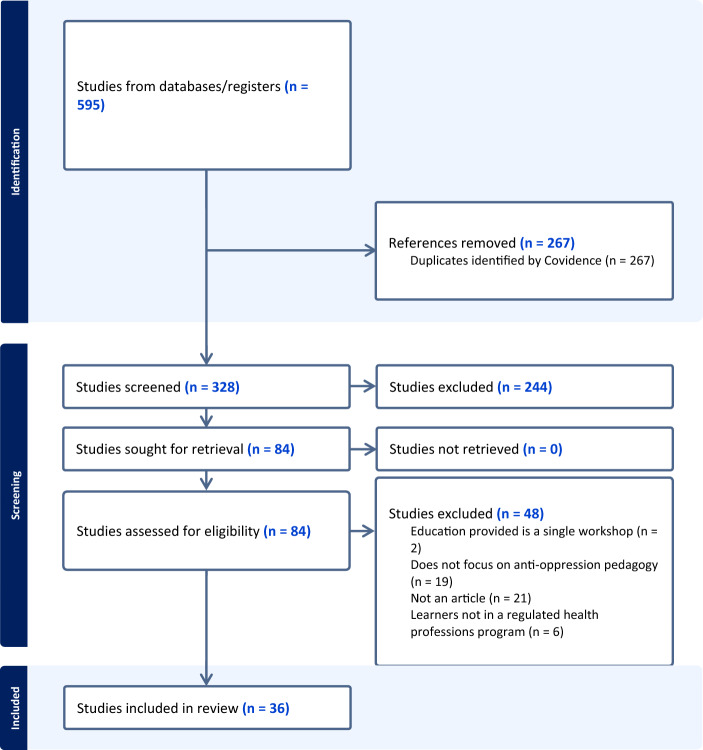
PRISMA flow diagram of review process

A total of 595 articles were retrieved from the search. After the removal of duplications, there were 328 articles eligible for screening by title and abstract. Once the screening of the titles and abstracts was completed, 84 articles were included for full review. Upon discussion between the first and second authors, and a second screening of the full articles, there were 36 articles included in the scoping review. Figure 1 depicts the review process using a PRISMA flow diagram.

### Descriptors of included articles

**Fig. 2:**
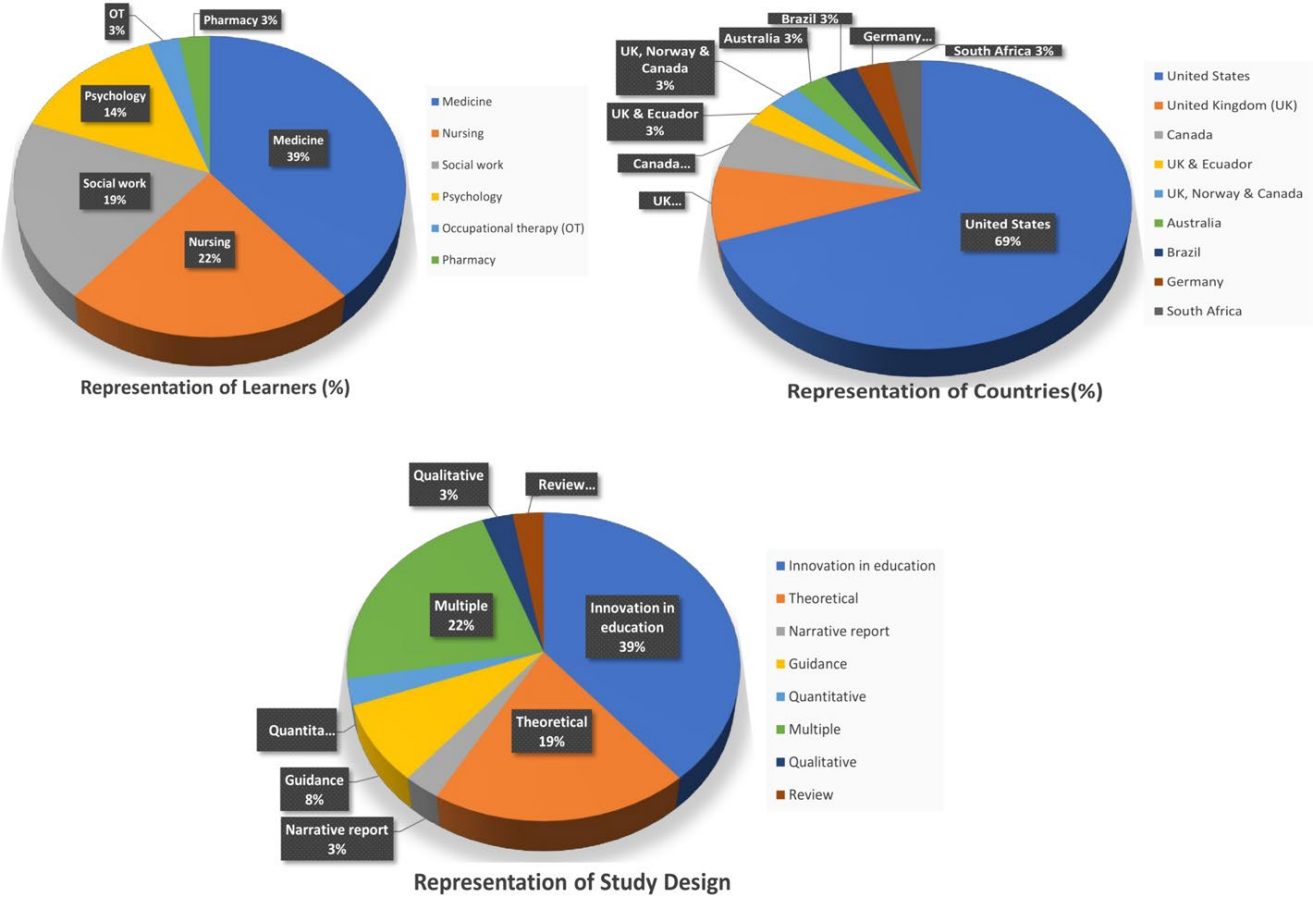
Depiction of health professional learner, study design, and country representation

Of the 36 articles, 14 provided education to medical learners (i.e., medical students and/or residents), eight to nursing learners, seven to social work learners, five to psychology learners, one to occupational therapy learners, and one to pharmacy learners. See Appendix for a summary of critical information on the key findings from each article. Most of the articles were from the Global North with one article combining the perspective of the Global North and Global South (Castro Romero & Capella Palacios, 2020), one article from the Global South (Carrijo et al., 2022), and one article from the Global North was based on a study from the Global South (Simaan, 2020). Specifically, 25 articles were published in the United States, three in the United Kingdom (UK), two in Canada, and two in the UK and another country (i.e., UK and Ecuador and UK, Norway, and Canada). The remaining four articles were published in the following countries: Australia, Brazil, Germany, and South Africa. The study design varied with most articles classified as an innovation in education, which was defined as creating and dispersing new educational tools and instructional practices and changing processes and practices to improve quality (Foray & Raffo, 2014). Seven articles were theoretical, eight articles utilized ‘multiple’ designs, and three articles provided guidance or recommendations. The remaining four articles each had a different study design. Figure 2 provides visual representation of learners, country of publication, and study design.

### Conceptualization of anti-oppression pedagogy

Upon review of the articles, ‘anti-oppression pedagogy’ was not widely used or conceptualized within health professions education. However, there were some engagements with the concept or related constructs, and anti-oppression was also found to be conceptualized and implemented in important ways. AOP was explicitly conceptualized in only three articles using the terms ‘anti-oppressive pedagogy’ (Punchhi et al., 2022), ‘antioppressive education’ (Power & Sistare, 2021) and ‘antioppressive frameworks’ (Bower et al., 2021). In addition, a few articles indirectly conceptualized AOP. These articles did not use the term AOP, but the described curriculum focused on anti-oppression. This suggests that these authors (Blanchet Garneau et al., 2018; Holley et al., 2015; Hutchison, 2015; Roy et al., 2022; Stahl et al., 2022) were indirectly conceptualizing AOP.

Punchhi et al. (2022) conceptualized AOP as a shift to understanding the root causes of inequities and a framework that “centres oppression within the healthcare system” (p. 567). Furthermore, implementing AOP requires “foundational and historical knowledge of oppression to develop a theoretical understanding of anti-oppression” (p. 571). Power and Sistare (2021) conceptualized anti-oppression education as resisting and challenging oppression and injustice at the micro and macro levels utilizing a variety of theoretical approaches including critical theory, feminism, and post-colonial perspectives. Bower et al. (2021) conceptualized anti-oppression pedagogy as anti-oppressive frameworks, which are based on the work of Zinga and Styres (2018). Anti-oppressive frameworks are used to teach learners to go beyond identifying oppression to critically analyze potential solutions to address the underlying social injustices. Anti-oppressive frameworks also facilitate critical consciousness (Bower et al., 2021).

An additional five articles (Blanchet Garneau et al., 2018; Holley et al., 2015; Hutchison, 2015; Roy et al., 2022; Stahl et al., 2022) indirectly conceptualized AOP. Blanchet Garneau et al. (2018) proposed a critical antidiscriminatory pedagogy to provide guidance on enacting social justice in practice and education. The terms ‘anti-discriminatory’ and ‘anti-oppression’ are used interchanageaby (Harlow & Hearn, 1996). This suggests that Blanchet Garneau et al. (2018) proposed a critical antidiscrimatory pedagogy which was conceptualized as “a framework for health care professionals to analyze power relations at multiple levels and in multiple intertwined contexts to look at and disrupt structural and individual-level dynamics producing and reproducing systemic discrimination” (p. 4). Thus, critical anti-discrimination pedagogy is based on a critical intersectional perspective of discrimination and it aims to cultivate transformative learning (Blanchet Garneau et al., 2018). Holley et al. (2015) referred to oppression frameworks which they described as used to “understand the systemic nature of racism, sexism, and other forms of oppression” (p. 399). Oppression frameworks contibute to reflection on privilege and one’s role in sustaining and tackling systems of oppression (Holley et al., 2015). Therefore, AOP was indirectly conceptualized as utilizing oppression frameworks.

Moreover, Hutchinson (2015) described a conceptual framework for teaching that provided a more comprehensive way to disrupt oppression. This implied that this framework indirectly conceptualized AOP. The framework consisted of anti-oppressive practice and theory along with reflexive lifeworld approaches to tackle oppression (Hutchinson, 2015). Anti-oppressive practice and theory challenge and aim to address structural inequalities. A lifeworld-led approach focuses on individuals’ everyday experiences which are marginalizing and isolating and the power of the individual to alter their lives. Reflexivity recognizes one’s own biases and the potential impact of this bias (Hutchison, 2015). Therefore, AOP was conceptualized as reflecting on one’s biases, focusing on individuals’ experiences and abilities, and learning to resist and mitigate structural inequalities.

Roy et al. (2022) and Stahl et al. (2022) referred to their currciula as anti-oppressive curricula and an anti-oppression capstone course, respectively. This implied that the described curriculum conceptualized AOP even though the term was not defined or discussed. Roy et al. (2022) described an anti-oppressive curriculum that incorporated the concepts of justice, equity, diversity, and inclusion, coupled with using a critical race theory framework to guide this curriculum. Stahl et al.’s (2022) anti-oppression capstone course utilized anti-oppressive pedagogical approaches such as decision case analysis and theatre of the oppressed, which incorporated learning about and discussing anti-oppressive approaches via acting. Therefore, AOP was conceptualized as teaching about anti-oppressive concepts and utilizing anti-oppressive approaches.

### Conceptualizations aligning with anti-oppression pedagogy

On review of the articles, a few did not conceptualize AOP but described constructs that aligned with AOP (Castro Romero & Capella Palacios, 2020; Froggett et al., 2015; Williamson et al., 2022). Though these articles did not report a focus on anti-oppression, the education provided had similar aims as AOP. In this way, these articles aligned with AOP and expanded the understanding of AOP. Castro Romero and Capella Palacios (2020) described a curriculum that was liberatory, decolonial, and examined power and oppression using forum theatre. Similar to theatre of the oppressed, in forum theatre, learners worked in small groups to develop an issue from everyday life that they enacted in a play allowing the ‘oppressor’ to win. The play was repeated and the spectators, who were also learners, interceded to change the outcome. Afterwards, the class reflected on the recommended solutions. Forum theatre and theatre of the oppressed teaches learners about and addressing oppression (Castro Romero & Capella Palacios, 2020). Froggett et al. (2015) highlighted challenges with anti-oppressive theory in that, alone, it may result in formulaic thinking versus understanding the complexity of oppression (Froggett et al., 2015). The authors described a course that utilized a psychosocial perspective of oppression to better facilitate understanding oppression. The course included a focus on racism, gender discrimination, and other forms of oppression from the psychosocial perspective. Therefore, this course aligned with AOP and suggested that it was conceptualized as not simply anti-oppressive theory but the incorporation of a psychosocial approach to support learners in understanding how oppression is “produced societally and institutionally, how it is enacted in interpersonal relationships and also how it is internalized, represented, and reproduced by individuals within any given set of power relations” (Froggett et al., 2015, p. 144).

Williamson et al. (2022) conceptualized equity literacy. Equity literacy was defined as the “the ability to recognize, respond to, redress, cultivate, and sustain efforts to disrupt inequities in various contexts of action and at multiple scales” (Williamson et al., 2022, p. 2). It was conceptualized in the medical context as recognizing health inequities and contributing factors, developing the skills to respond to inequities in patient care, acquiring knowledge to redress systemic inequities, and learning to cultivate and sustain efforts to disrupt inequities in health care (Williamson et al., 2022). This conceptualization aligns with the aims of AOP and suggests that equity literacy draws on AOP.

In addition, two articles focused on a specific form of oppression and the alignment with AOP varied. Edwards et al. (2023) focused on teaching about disability via authentic case study scenarios. The cases included themes on stigma, discrimination, gender, and sexuality. In addition, this course taught learners to identify the structural drivers of social exclusion, to utilize strategies to empower people living with disability, and to break down barriers related to discrimination. This demonstrates that curricula focused on one system of oppression may teach about multiple forms of oppression and may align with AOP. Keuroghlian et al.’s (2022) curriculum focused on the lesbian, gay, bisexual, transgender, queer, intersex, asexual, and all sexual and gender minorities (LGBTQIA+) community. The recommendations for this curriculum included utilizing a social determinants of health framework, which focused on understanding and addressing health inequities and the intersection of inequities (Keuroghlian et al., 2022). A curriculum with this focus may align with AOP and suggests that the implementation of the curriculum impacted whether it aligned with AOP.

### Focus on antiracism

Most of the articles focused on antiracism in ways that overlapped with the description of AOP in the inclusion criteria. Although the curricula described in these articles did not focus on anti-oppression, the curricula achieved the goals of AOP through teaching about anti-racism. This suggests that conceptualization of AOP may include antiracism. Three articles specifically focused on antiracist education (Altman et al., 2021; Balhara et al., 2022; Ona et al., 2020). Altman et al. (2021) described antiracist education as facilitating the identification and critical analysis of racialized power relations, the understanding of race as a social construct, and the exploration of intersecting systems of oppression. Antiracism pedagogy “centers the experiences of marginalized communities; acknowledges the interconnection of individual experience to larger systems of power, privilege, and oppression; and aims to transform these systems” (Altman et al., 2021, p. 285). Similarly, Balhara et al. (2022) indicated that antiracist education and antiracist pedagogy address racism and promote critical reflection about racialized power relations; one’s own social position, biases, and privileges; and contributors to racism. Ona et al. (2020) added to this by exploring antiracism curricula, which teaches about structural racism including the history of racism and the ways power, privilege, and policy uphold racism. These conceptualizations overlapped with AOP in the manner that they taught about power dynamics, privilege, and root causes of oppression. In addition, Ona et al. (2020) mention that anti-oppression curricula “needs to occur earlier and more broadly” (p. S163), which suggests that AOP should explicitly include antiracism.

Antiracism was specifically conceptualized as reflection on structural racism (Carrijo et al., 2022), resisting oppressive structures (Koch, 2021), dismantling health disparities (Robinson et al., 2021), and a 4-step framework to see, name, understand, and to act (Solomon et al., 2021). Curricula based on this understanding of antiracism had the potential to overlap with AOP depending on its implementation. For example, curricula which overlapped with AOP elicited reflection on structural racism (Carrijo et al., 2022), required discussion and reflection on social inequities and systemic racism (Koch, 2021), and discussed constructs of racism and other forms of oppression (Robinson et al., 2021). Solomon et al. (2021) did not provide a curriculum, but the 4-step framework was considered in the development of Balhara et al.’s (2022) curriculum, which demonstrated this framework can be utilized to overlap with AOP. Similarly, the curriculum in the work of Lord et al. (2023) aimed to increase antiracism and overlapped with AOP by increasing the understanding of systemic racism. This curriculum was based on transformative pedagogy and critical consciousness (Lord et al., 2023).

The articles that focused on antiracism and cultural awareness (Carter & Phillips, 2021) and antiracism and structural competency (Afolabi et al., 2021; Carter & Phillips, 2021; Godley et al., 2020; Wear et al., 2017) had similar conceptualizations of antiracism. The described curricula also overlapped with AOP. Curricula related to antiracism and cultural awareness included courses that provided learners with a deeper understanding of race, racism, and other forms of oppression such as ability status (Carter & Phillips, 2021). Curricula focused on antiracism and structural competency included understanding the influence of structural forces such as racism and sexism (Afolabi et al., 2021); providing context for discussions about racism and structural inequality (Godley et al., 2020); and exploring issues of power, privilege, and oppression (Wear et al., 2017). These articles highlighted the overlap and similarities in teaching about anti-racism and anti-oppression. Coleman’s (2020) work which focused on racial justice also overlapped with AOP by considering intersectionality and recommending a power and privilege course.

O’Neill and Miller (2015) had a similar conceptualization of antiracism. However, this article also critiqued anti-oppressive practice for decentralizing race and racism. In this curriculum, an explicit commitment to antiracism was selected to emphasize race and racism. One of the courses in this curriculum provided an overview of multiple forms of oppression and included the dynamics of privilege and oppression. The other course facilitated an understanding of racism at the micro, mezzo, and macro levels (O’Neill & Miller, 2015). The concepts discussed in these courses overlapped with AOP and suggest that AOP should include an explicit focus on racism.

Lemieux et al. (2021) and Drum et al. (2021) described curricula with potential to overlap with AOP, however they did not define or explicitly conceptualize antiracism. Lemieux et al. (2021) described a curriculum aimed to increase awareness of systemic racism which also overlapped with AOP. The curriculum was an intersectional anti-racist journal club; the club supported learners to recognize and address one’s internal racism and bias, to identify the ways institutions cause racial oppression; and to consider solutions (Lemieux et al., 2021). Drum et al. (2021) referred to a curriculum that may have aligned with AOP depending on the way the concepts were defined and the curriculum implemented. For example, one goal of antiracism teaching in Drum et al. (2021) was to equip residents with antiracism knowledge and skills. However, the description was lacking and thus overlap with AOP is unclear.

### Focus on decoloniality

Another area of focus of the reviewed articles was decoloniality which also overlapped with AOP. Similar to the articles on antiracism, these articles achieved the goals of AOP through teaching about decoloniality. This suggests that the conceptualization of AOP can include utilizing a decolonial approach and teaching about decoloniality. In this review, four articles utilized a decolonial approach to education (Blanche et al., 2021; Dutta, 2018; Mbaki et al., 2021; Simaan, 2020) and three articles taught about decoloniality (Fernandez, 2018; Hendrick & Young, 2018; Watkins et al., 2018). Blanche et al. (2021) utilized a decolonial lens to revise courses. A decolonial approach to teaching involved being reflexive, relying on participatory teaching strategies to avoid a top-down pedagogy, and centering knowledges that have been suppressed (e.g., Indigenous knowledge and practices). Integrating this approach into courses led to learners considering how knowledge is produced; reflecting on the experience of historical, class and gender oppression; and reviewing theoretical resources on colonialism, globalization, and decoloniality (Blanche et al., 2021). Dutta (2018) focused on decolonizing the curriculum, which disrupts and dismantles the normative European-American point of view and imagines alternative points of view. Decolonizing the curriculum led to classroom teaching that included recognizing intersecting forms of oppression and critically thinking about the role of institutions in oppression (Dutta, 2018). Simaan (2020) also focused on decolonizing a curriculum, which was conceptualized as emphasizing the work of non-Western, colonized writers and the knowledge of the colonized. This was implemented in a learning activity where learners reflected on and learned about the experience of a marginalized community (Simaan, 2020). Lastly, Mbaki et al.’s (2021) decolonizing efforts resulted in a framework that provided guidance on the diversification of the curriculum. This framework was based on decolonization and a decolonization narrative, which included the voices of marginalized individuals considering age, ability, gender, religion, and sexual orientation. Ways to incorporate this framework in teaching included increasing learners’ understanding of colonialism and discussing the impact of ‘othering’ and structural inequality (Mbaki et al., 2021). The concepts learned in these articles (Blanche et al., 2021; Dutta, 2018; Mbaki et al., 2021; Simaan, 2020) overlapped with AOP—this finding indicates that a decolonial approach to education may overlap with AOP depending on the way it is implemented.

Similarly, teaching about decoloniality overlapped with AOP. Fernandez (2018) utilized an assignment to facilitate learner’s decolonial thinking, which is “characterized by a critical consciousness of how racialized coloniality produced interconnected systems of oppression” (p. 295). Through this assignment, learners had the opportunity to learn about colonialism and systems of oppression. Hendrick and Young (2018) developed a framework for teaching about decoloniality. This framework can be used to teach learners about perspectives, explanatory theory, and intervention theory, which include a focus on Indigenous knowledge to justify practice decisions (Hendrick & Young, 2018). Watkins et al. (2018) integrated decoloniality in a decolonial curriculum to centre historically silenced knowledges. This article described anti-racist/decoloniality curriculum as acknowledging and contextualizing colonialism and the history of inequity; contextualizing the deconstruction of patriarchy, racism, and marginalization; and integrating non-Western and Indigenous philosophies and approaches (Watkins et al., 2018). The concepts in the anti-racist/decoloniality curriculum overlapped with AOP and demonstrated alignment in anti-racist, decolonial, and anti-oppression pedagogy.

### Integration of content

Given the significance of implementation, it is important to understand the integration of AOP and pedagogy that aligns or overlaps with AOP within health professions education. Throughout the articles, the content of curricula aligned with the purpose and the main concepts or constructs on which the content was based. For example, the purpose of the curriculum described by Bower et al. (2021) was to teach about the root causes of social inequities and to develop skills to dismantle systems of oppression. This curriculum was based on anti-oppressive frameworks and the content of the curriculum included learning about anti-oppressive frameworks; applying anti-oppressive frameworks to community-based projects; and enhancing the understanding of power, privilege, positionality, intersectionality, and oppression (Bower et al., 2021). As another example, Balhara et al. (2022) developed a health humanities curriculum (i.e., health humanities include transdisciplinary areas of study including arts, humanities, and social sciences) focused on antiracism. This curriculum was based on antiracist education and pedagogy and included learning about the history of racism, microaggressions, and the history of xenophobia in pandemics; discussing structural racism; and developing skills to respond to racist acts (Balhara et al., 2022). These examples demonstrate the ways the purpose, main concepts or constructs, and their integration aligned. In addition, the content often included teaching learners about power, privilege, and the structure or root causes of oppression; facilitated critical reflection and a consideration of one’s own position in systemic oppression; and helped learners to address structures and systems of oppression.

The content was most frequently delivered within courses. Several articles also delivered content through participation in specific activities. This included participating in practical experiences (Bower et al., 2021; Punchhi et al., 2022; Wear et al., 2017), designing and implementing a writing workshop for individuals residing in jail (Power & Sistare, 2021), and completing a family portrait assignment requiring white learners to reflect on the history of immigration and migration in their family compared to communities of Colour (Fernandez, 2018). A variety of strategies were utilized to teach the content and often multiple strategies were used in delivery. The most common strategies were discussions followed by cases involving discussion. Several articles also included the use of lectures, reflection activities, and learning through lived experiences or current events. However, Punchhi et al. (2022) found that the integration of AOP tends to have limited opportunities for hands-on, community-based learning and there is a misalignment of the dominant values and priorities of medical education and training with the key principles of anti-oppression.

A small group of articles focused specifically on utilizing the humanities. The use of the humanities in health professions education assists learners to “enhance empathy, perspective-taking, and openness to different viewpoints, and to prompt reflection of self, others and the world” (Kumagai & Wear, 2014, p. 973), which makes it ideal to support anti-racist or social justice learning in health education. The use of humanities within the small group of articles included Forum Theatre where learners had to develop and enact scenarios in which the oppressor wins then the audience enacts potential solutions in the reenactment of the scenario (Castro Romero & Capella Palacios, 2020; Stahl et al., 2022). The use of humanities also included the incorporation of art, short stories, poetry, books, and podcasts into teaching (Balhara et al., 2022; Godley et al., 2020; Wear et al., 2017). Less frequent strategies included online modules (Punchhi et al., 2022; Roy et al., 2022; Williamson et al., 2022), a journal club (Afolabi et al., 2021), and simulation activities (Williamson et al., 2022).

## Discussion

This scoping review provides an overview of 36 identified articles to explore the conceptualization and integration of AOP in health professions education. When exploring AOP, it is relevant to consider the location of the articles to understand the voice and knowledge represented in the literature. Most of the articles were from the Global North, which highlights the emphasis of this perspective in the literature related to AOP. This is reflective of Blanche et al.’s (2021) review of the 2018 special issue of the American Journal of Community Psychology and the 2017 special issue of the South African Journal of Psychology, which highlighted that almost all contributions originated from the Global North.

AOP was conceptualized in eight articles. This indicates that AOP is not common terminology within health professions education. There was not one way in which AOP was conceptualized but there were similarities. In these articles, AOP was consistently conceptualized as including an understanding of the social structures and root causes of oppression, critically reflecting on oppression and privilege, and often learning to resist or disrupt oppression. AOP may also be conceptualized as forms of teaching such as Stahl et al.’s (2022) description of an anti-oppression course in which learners participated in decision case analysis and theatre of the oppressed.

A few articles did not conceptualize AOP, but the purpose of the curriculum and the discussed concepts aligned with AOP. These articles expanded the understanding of how AOP may be conceptualized in health professions education. Williamson et al. (2022) utilized an equity literacy framework to teach learners to recognize, respond to, redress, and disrupt health inequities including systemic oppression. This suggests that AOP may be conceptualized as an equity literacy framework. Froggett et al. (2015) critiqued anti-oppressive practice for not including a focus on how oppression is internalized. A course was developed that supported learners in understanding how the social world is internalized and focused on the psychosocial configuration of systems of oppression. This indicated that AOP was conceptualized as going beyond anti-oppressive practice to focus on how oppression becomes internalized.

The conceptualization of AOP was further expanded by a focus on antiracism. Most articles focused on antiracism in a manner that overlapped with AOP. This aligns with Corneau and Stergiopoulos (2012) who highlighted that both anti-racism and anti-oppression philosophies emphasize issues of power imbalances and the importance of self-reflexivity. Furthermore, an anti-racist framework was described as a strategy within the spectrum of anti-oppression practices (Corneau & Stergiopoulos, 2012) and anti-racism and anti-oppression both aim to identify, challenge, and change the structures that perpetuate systemic racism and other forms of oppressions (Corneau & Stergiopoulos, 2012; Dei, 2000). Similarly, this scoping review found articles that focused on antiracism overlapped with AOP by teaching learners about power, privilege, and the root causes of racism and often other forms of oppression. This suggests that in health professions education, antiracism is utilized to teach specifically about anti-racism and more broadly about anti-oppression. One article in this review considered an anti-oppression versus an antiracism commitment and selected the latter because the authors determined that racism was underemphasized while other forms of oppression were discussed in the program (O’Neill & Miller, 2015). This article also critiqued anti-oppressive practice for decentralizing racism (O’Neill & Miller, 2015), which highlights a potential benefit to conceptualizing AOP in a way that centers antiracism. Blanchet Garneau et al. (2018) further supported this by proposing a critical antidiscriminatory pedagogy, which addressed the systemic and structural nature of multiple forms of oppression including racism, ageism, and gender inequities. Roy et al. (2022) also highlighted the importance of intentionally teaching the context and history of racism and oppression.

The articles that focused on decoloniality similarly overlapped with concepts of AOP. This echos Zinga and Styres (2018) who stated that both decolonizing and anti-oppressive pedagogies resist the mainstream approaches to education and challenge assumptions that are embedded in the status quo. Decolonizing pedagogies loosely fit in the broader area of anti-oppression (Zinga & Styres, 2018). Kalbarczyk et al. (2023) also highlighted that teaching anti-oppression principles is an approach to decolonizing education. The articles in this review demonstrated that both teaching about decoloniality and decolonial teaching overlapped with AOP through teaching learners about colonialism, decolonization, and Indigenous and non-Western knowledge. In learning about these concepts, learners acquired knowledge about power imbalance and privilege further overlapping with AOP.

Overall, there is not a clear or consistent conceptualization of AOP in health professions education. This is reflective of literature on AOP that varied in describing this form of pedagogy (Aqil et al., 2021; Dalrymple & Burke, 1998; Galloway et al., 2019; Jakubec & Bourque Bearskin, 2020; Kumashiro, 2000; Lavallée, 2014; Lyons et al., 2023). Despite the variation, AOP was generally conceptualized as a broad concept that facilitated the understanding, critical reflection on, and action against structural and systemic forms of oppressions. In addition, there was variety in the way AOP was integrated in health professions education. The content typically aligned with the aim of the curriculum, and the concepts on which it was based. Clarity in the content delivered is required to demonstrate whether the content aligned or overlapped with AOP. For example, anti-racist constructs were delivered in sessions on antiracism (Drum et al., 2021) but it was not clear if these constructs were delivered in a manner that taught about power, privilege, or the causes of racism, or simply acknowledged racism and anti-racism.

Throughout the articles, the delivery methods and teaching strategies varied. The most common teaching strategies were discussions, cases, reflection, learning through lived experiences, and the incorporation of humanities. The strategies suggest a potential benefit to participating in activities to facilitate learning. This includes participating in theatre of the oppressed (Stahl et al., 2022); an olive farming activity based on knowledge which was developed in the Global South (Simaan, 2020); and an assignment in which one’s family is interviewed to explore the history, or lack of history, of immigration and migration (Fernandez, 2018). A longitudinal curriculum seemed preferential to stand alone sessions in facilitating learning. The variety in content, delivery methods, and teaching strategies indicated that there was not yet a clear way to integrate AOP in health professions education.

Based on this review, health professions education conceptualized antiracism and decoloniality in ways that overlapped and were part of the broad term AOP. However, the extent to which these concepts overlapped with AOP often depended on their implementation. To overlap with AOP, implementation could not simply acknowledge colonialism or racism but needed to facilitate understanding and reflection on structures of oppression, power, and/or privilege. When integrating AOP, institutions should also be challenged to examine a commitment to anti-oppression at all levels of the program since this influences the delivery and impact of the curriculum (Mbaki et al., 2021; O’Neill & Miller, 2015; Punchhi et al., 2022). This includes considering the impact of the diversity of the learners, faculty, and leadership (O’Neill & Miller, 2015); the inclusion and compensation of experts in anti-oppression (Afolabi et al., 2021); the need for faculty development; the opportunity to learn from communities with lived experience; the admissions process (Punchhi et al., 2022); the institutional policies; and the engagement of diverse stakeholders in the development and implementation of the curricula (Mbaki et al., 2021). These considerations align with Manchanda et al. (2023) who highlighted similar obligations of educators and leadership to address and teach about health inequities and oppression.

### Implications for health professions education

This scoping review provides insight on conceptualizing and incorporating AOP in health professions education. AOP may be utilized in health professional programs to facilitate understanding the intersection of multiple forms of oppression, the systems and structures that contribute to health inequities, and ways to resist and tackle forms of oppression to lead to better patient health outcomes. Considering a broad approach that includes a focus on antiracism, decoloniality, intersectionality, systems and structures of oppression, and a critical lens is important in the conceptualization of AOP. Health professional programs should also consider incorporating AOP in a longitudinal manner and integrating components of discussions, cases, reflection, learning through lived experiences, and humanities to facilitate learning. In addition, health professional programs should further explore the conceptualization and integration of AOP. For example, health professional programs should share the ways that AOP is taught to develop a greater understanding of AOP and the delivery methods that most effectively facilitate learning.

### Gaps and future research directions

Despite the benefits of AOP, this scoping review demonstrated that AOP is not commonly utilized terminology in health professions. It revealed that concepts that align or overlap with AOP are more commonly utilized and conceptualized than AOP. Further research on the benefits of AOP in health professions can demonstrate the need for health professions to include AOP in curricula. A clear and consistent conceptualization of AOP will also aid programs in understanding how to include AOP in curricula since it is challenging to include if it is not well defined. This would also help to determine when programs should include AOP in curricula and facilitate communication and collaboration amongst programs to enhance AOP in health professions education.

Educators would also benefit from further research on the evaluation of AOP curricula and the assessment of learners. For example, several studies evaluated the described curriculum via learners’ evaluation of the experience (Afolabi et al., 2021; Edwards et al., 2023; Froggett et al., 2015; Godley et al., 2020; Keuroghlian et al., 2022) and Drum et al. (2021) utilized a Likert scale to evaluate the value of the learning experience. Assessment methods varied and included formative assessments such as critical reflection activities (Williamson et al., 2022), formative and summative short reflective writing assignments (Altman et al., 2021), in-class presentations (Bower et al., 2021), simulation exams (Carter & Phillips, 2021), and exam questions focused on topics such as culturally sensitive care (Carter & Phillips, 2021). The evaluation and assessment methods are beyond the scope of this review, but this research would support educators in better understanding how to effectively evaluate the education provided and the impact on learners.

In addition, AOP and the integration of AOP in curricula needs to be further explored. There were a variety of teaching strategies utilized without direction on the most effective methods of integration. This review indicates that there were common teaching methods, but there was no evidence that a specific teaching method resulted in enhanced learning. There was also increasing literature on the utilization of humanities, which needs to be further explored. Overall, more research and evaluation need to be conducted to determine teaching strategies that help learners understand and resist systems and structures of oppression and promote changes in practice to improve health outcomes for a diverse patient population.

While the aim of this scoping review was to assess if and how anti-oppression pedagogies was conceptualized and integrated in health professions education, a deeper focus on the broader context of academic institutions fundamentally shifting their governance, policies, and practices to *be* anti-oppressive and anti-racist is worth further study. Such research would be timely for two reasons. First, with advent of Equity, Diversity, Inclusion, and Access policies across academic institutions this future research could illuminate how such initiatives are, or are not, addressing oppression and racism. Second, Indigenous and Black scholars have recently drawn attention to the inherent colonial and racist discourses and practices of academic institutions (Brunette-Debassige, 2023; Henry et al., 2017). As such, future research could bring more attention to the complexities of advancing anti-oppression pedagogy in academic institutions imbued with ongoing colonialism and racisms.

### Limitations

A few limitations were identified in this study. The search strategy focused on the following key terms: anti-oppression, antiracism, and decoloniality. Articles that described curriculum that taught about oppression but did not include these key terms were not included in this paper. Consequently, relevant articles that focused on additional concepts aligned with AOP may not have been identified. In addition, the search was restricted to three electronic databases from 2015 to 2023. As a result, relevant articles in other databases and prior to 2015 were not included in this study. Therefore, articles may have been missed that would have further expanded the understanding of AOP and provided additional methods for integrating AOP in health professions education. This review also excluded non-English articles which may have skewed the findings to articles from the Global North. Lastly, there may be political risks to labelling one’s scholarship and/or teaching as anti-oppressive, which may limit the number of scholars willing to publish about their anti-oppressive pedagogies. For example, teaching about critical race theory and anti-oppression in Republican states within the United States of America has been silenced, shunned, and in some cases regulated by law (Goldberg, 2023; Schwartz, 2021). Whereas in Canada, multiculturalism and anti-racism are supported by the Government of Canada through the Canadian Multiculturalism Act, (1985) and supported through anti-racism government programs (Department of Canadian Heritage, 2024).

## Conclusion

AOP facilitates understanding and addressing the structures and systems of oppression which cause health inequities. There is an increasing call for AOP to be incorporated in health professional programs to reduce health inequities and improve patient care and health outcomes. This scoping review indicates that AOP is not commonly utilized terminology in health professions education as only three studies clearly utilized an anti-oppression pedagogy in their articles In addition, AOP is not conceptualized in one consistent manner but is generally viewed as a broad concept that focuses on antiracism; decoloniality; intersectionality; and supporting learners to understand, critically reflect on, and act against structural and systemic forms of oppressions. Thus, this review suggests that employing a diverse range of constructs related to teaching about anti-oppression may lead educators to work in isolated silos. A strength of this review is that it highlights the need for health professions education to develop one clear concept that educators use when teaching about anti-oppression, which may reduce working in silos and allow educators to better collaborate with each other in advancing this work.

There is variation in the integration of AOP in health professions education with the most common methods consisting of discussions, cases, reflection, learning through lived experiences, and the incorporation of humanities within a longitudinal curriculum. Further research on AOP is required to emphasize the benefits, provide clarity on the conceptualization, and determine the most effective methods of integration to better support programs in including AOP in the curricula. Based on this review, health professional programs should consider incorporating AOP in curricula with a broad approach that focuses on antiracism, decoloniality, intersectionality, systems and structures of oppression, and a critical lens. In addition, health professional programs should consider a longitudinal AOP that utilizes discussions, cases, reflection, learning through lived experiences, and humanities to facilitate learning. Health professional programs can also examine their commitment to anti-oppression in all aspects of the program. The aim of AOP is to promote change in practice and improve health outcomes for patients. Therefore, there is a benefit to including and further researching AOP in health professions education.

## Appendix

Summary of articles included in the scoping review

**Table:** 

Authors	Year	Country	Study design	Learners	Purpose	Main concepts	Conceptualization of AOP	Curriculum description	Integration
Afolabi et al.	2021	United States	Innovation in education; create new curricula	Medical students	Highlight examples of student-led efforts to advance anti-racist curricula	An anti-racist lens equips trainees to recognize and address structural inequities. Structural competency considers how disease is influenced by social context & social context is influenced by structural forces	AOP is not conceptualized Anti-racism and structural competency overlap with AOP	Two separate 3-week courses related to race and other social determinants of health including structural competency training curriculum	Community members with relevant lived experience; placing inequities in the context of structural violence; drawing on more challenging reading materials and discussion questions
Altman et al.	2021	United States	Innovation in education; new approach to case-based learning	Nursing students	Describe a process for creating an antiracist environment for case-based learning	Antiracist education helps learners identify and critically analyze racialized power relations, understand the social construction of race, and examine the interconnecting systems of oppression Antiracist pedagogy acknowledges that race is a social construct and frames race and racism as part of larger systemic and structural forces	AOP is not conceptualized Antiracist education and antiracist pedagogy overlap with AOP	Integrating positionality into case-based learning Coursework on social determinants of health, systemic racism, implicit bias, and intersectionality prior the case-based learning	Case based learning; modeling positionality; students reflecting on their own identity and positionality; exploring power dynamics in the case
Balhara et al.	2022	United States	Innovation in education; create new curriculum	Residents and medical students	Create a health humanities curriculum focused on antiracism	Antiracist education includes critical reflection about, and subsequent action on, the impacts of power relations intrinsically tied to race Antiracist pedagogy interrogates the past, reflects on the present, and prepares for the future and requires a critical consciousness of one’s biases and privileges	AOP is not conceptualized Antiracist education and antiracist pedagogy overlap with AOP	Health humanities-based longitudinal curriculum comprised of 8 weekly didactic sessions and an asynchronous speaker series	Narrative medicine; relevant visual art, literature, poetry, nonfiction, and podcasts paired with exploring and deconstructing past or current racist structures or practices; engaging in discussion with patients
Blanche et al.	2021	South Africa	Innovation in education; revise modules	Community psychology students	Present the recirculation of three modules using a decolonial lens	Decolonial theory attempts to historically situate societies and communities Decolonial approach includes becoming aware of one’s own positioning and complicity in the colonial epistemic project	AOP is not conceptualized A decolonial approach overlaps with AOP	Three courses consisting of an honors course, a second-year course, and a third-year course	Focus on how knowledge is created and reproduced; reflection on the social and subjective phenomena that students encounter; theoretical resources on topics such as colonialism, postcolonialism, globalization, and decoloniality; theoretical readings and stories about varying experiences
Blanchet Garneau et al.	2018	Canada	Theoretical	Nursing students	Propose a critical antidiscriminatory pedagogy (CADP) to provide guidance regarding how to enact social justice	The CADP is grounded in a critical intersectional perspective of discrimination, aims at fostering transformative learning, and involves a praxis-oriented critical consciousness Transformative learning is using a prior interpretation to develop a new interpretation of one’s experience to guide future action	AOP is not explicitly conceptualized The CADP indirectly conceptualized AOP	Include the CADP in teaching (e.g., diabetes teaching)	Beyond the scope of this paper to provide specific strategies for implementing CADP in curriculum
Bower et al.	2021	United States	Innovation in education; develop new content	Nursing students	Describes the process to integrate critical service learning (CSL) pedagogy to teach students to understand the multilevel roots of social inequities and to dismantle unjust systems	Anti-oppressive frameworks help move students from the identification of harmful social conditions to critical analysis of possible solutions to mitigate the social injustices underlying the exposure CSL adds the dimensions of intentionally working toward social change, redistributing power, and developing authentic partnerships	AOP is conceptualized as anti-oppressive frameworks	CSL applied across four courses which included one didactic and three practicums offered across four semesters	Lecture and discussion; apply anti-oppressive frameworks to community-based projects using discussion-based case studies and written structured reflection activities to deepen student understanding of power, privilege, positionality, intersectionality, and current and historic oppression of populations
Carrijo et al.	2022	Brazil	Narrative report; descriptive and qualitative	Medical students	Discuss possible actions to combat racism as a root cause of health inequities	Structural racism results from the functioning of institutions that confer disadvantages and privileges based on race and produces immeasurable losses and inequities	AOP is not conceptualized Structural racism overlapped with AOP	Health of the Black population weekly course for fifteen weeks	Reflection on structural racism; lectures; provide content in various formats, such as scientific articles, books, and podcasts among other audiovisual resources; students carry out critical reviews
Carter & Phillips	2021	United States	Guidance based on the literature	Nursing students	Provides suggestions for curricular change to rebuild a curriculum that is nonbiased and inclusive	Cultural awareness and antiracism not defined. Curriculum must include infusion of components such as cultural awareness and responsiveness, the structural causes of racism, inequities, and implicit biases	AOP is not conceptualized Antiracism and cultural awareness overlapped with AOP	Example pathophysiology courses; stand-alone courses about equity and antiracism over the course of a year to equip learners with an understanding of racism, ability status, and gender identity	Infuse in classrooms, labs, and simulations; may describe inequalities in access and fair treatment across races; may explore care inequalities that may lead to health disparities in underrepresented clients
Castro Romero & Capella Palacios	2020	UK and Ecuador	Innovation in education; new approach in education	Community psychology students	Discuss the limitations and possibilities of engaging in a praxis that is decolonial and liberatory	A liberatory praxis critically examines our realities and transforms social reality Decolonising problematizes theories, methodologies & practices generated by the Global North	AOP is not conceptualized The implementation of Forum Theatre aligned with AOP	International dialogue and pedagogical exchange by interchanging Forum Theatre scenes between Ecuadorian and UK students	Cases using Forum Theatre
Coleman	2020	United States	Guidance based on the literature and experience	Nursing students	Explores nursing education as an upstream intervention to addressing racial inequities	An anti-racist position explicitly names and addresses the ideologies and culture of racism, oppression, and eurocentrism that permeate all institutions of education	AOP (AOP) is not conceptualized The recommendations for a racial justice praxis align with AOP	A curriculum was not described	Recommendations to adopt an explicitly anti-racist position; institute a power and privilege course with a foundation in racism and its history; integrate current events, and use journaling as a reflective practice
Drum et al.	2021	United States	Empirical; quantitative	Residents	Create an elective including anti-racism work	Anti-racism was not defined	AOP is not conceptualized Anti-racism work may align with AOP	An elective with two sessions on antiracism to develop anti-racism knowledge and skills	Training in anti-racist concepts, practice responding to simulated microaggressions; and reflection on inclusive practices
Dutta	2018	United States	Multiple—theoretical, qualitative, and guidance	Community psychology students	Disrupt dominant modes of knowledge production and imagine non-hierarchical possibilities	Decoloniality entails a fundamental transformation of the terms of knowledge production, striving to a new vision Decolonizing the curriculum disrupts and dismantles normativity of the Euro-American vantage point and nurturing capacities to imagine alternatives that are not predicated upon hierarchies of difference	AOP is not conceptualized Decolonizing the curriculum overlapped with AOP	A seminar which included a segment on critical social transformations and a segment focusing on methodological critiques	Recognizing differences along intersections of age, race, ethnicity, gender, ability, and socioeconomic class; incorporating critical insights about the wider institutional contexts in which differences are configured
Edwards et al.	2023	Germany	Multiple—innovation in education; develop new content and qualitative	Social work students	Evaluates problem-based learning and traditional teaching methods to deliver disability content	A socio-political framework focuses on the deconstruction of Global North, western, colonial understandings of disability and facilitates the identification of individual and structural drivers of social exclusion	AOP is not conceptualized The curriculum may align with AOP	Five-day elective course on disability within a global context	Problem-based learning; sessions covered themes such as disability as a social relationship, stigma, discrimination, gender, and sexuality; student presentations that synthesised work on the case studies and critical reflections
Fernandez	2018	United States	Innovation in education; develop new tool	Community psychology students	Demonstrate how the Family Portrait Assignment facilitated white student’s decolonial thinking and disrupting white innocence	Decoloniality contests the problematic structuring of a system that organizes and demarks people into categories, and forms of Othering that reify the oppression of generations and nations Decolonial thinking is critical consciousness of how racialized coloniality produced inter-connected systems of oppression	AOP is not conceptualized The approach to incorporating decolonial thinking overlaps with AOP	Introductory course in race and ethnicity with a Family Portrait Assignment	Interview family member(s); critically examine the sociohistorical and sociopolitical factors that shaped the family’s immigration experiences; identify systemic structures that impacted access to resources; comparing the family’s immigration story to those of other immigrant groups
Froggett et al.	2015	UK, Norway, and Canada	Innovation in education; develop new course	Social work students	Explores a course rationale, content, delivery, and assessment	The psychosocial perspective increases awareness of the psychological self while maintaining a socially critical stance. Anti-oppressive theory can lead to formulaic thinking versus grasping complexity	AOP is not conceptualized. A psychosocial perspective aligns with AOP	A course on use of self	Lectures; group and case discussion; use of events in the media; focuses on psychosocial configurations of racism, gender discrimination and other forms of marginalisation
Godley et al.	2020	United States	Multiple—innovation in education; develop new curricula and qualitative	Medical students	Piloted an educational program using art to address racism in medicine	Anti-racism is identifying and eliminating racism by critically evaluating and reforming systems, institutional structures, policies, and language, with the goal of redistributing power equitably	AOP is not conceptualized Anti-racism overlaps with AOP	“Can We Talk About Race?” is a curricular innovation included in a longitudinal mandatory curriculum in Social and Health Systems	Visit a museum, an hour-long discussion on a piece of art followed by a 30-min reflection
Hendrick & Young	2018	Australia	Theoretical	Social work students	Presents a framework for teaching about decoloniality	Attempts to avoid binaries between coloniality and decoloniality to offer opportunities to mediate between them. The framework includes considering perspectives (e.g., human rights); explanatory theory (e.g., critical theories, healing theories); intervention theory (e.g., anti-discrimination); and decolonising social work	AOP is not conceptualized The framework for teaching about decoloniality may overlap with AOP	A curriculum was not described. An example case was provided	Use the framework to answer the question, if you were assigned to this family, what would you do, and why?”
Holley et al.	2015	United States	Theoretical	Social work students	Provides a theoretical framework and strategies to teach about oppression and privilege	Oppression frameworks facilitate understanding the role of history in creating current circumstances, intersections of diversity, the relationship between diversity and oppression, internalized oppression and privilege, and the systemic maintenance of oppression	AOP is not explicitly conceptualized Oppression frameworks indirectly conceptualized AOP	A curriculum was not described	Harro’s (2000) cycle of socialization is an approach that can facilitate understanding systemic oppression related to multiple forms of difference
Hutchison	2015	UK	Theoretical	Nursing students	Outline an anti-oppressive practice model and a reflexive lifeworld-led approach to teach to challenge oppressive structures or behaviours	Anti-oppressive practice challenges structural inequalities; criticized for focusing primarily on the structural understandings of power Lifeworld-led care involves a philosophy of the person Anti-oppressive practice and a reflexive lifeworld-led approach offer a more comprehensive framework to tackle oppression	AOP is not explicitly conceptualized Anti-oppressive practice and a reflexive lifeworld-led approach indirectly conceptualized AOP		Vignettes that illustrate a variety of everyday patient experiences; reflective exercise to see the interactions between nurses and patients that can be oppressive; provide a fundamental grasp of the key sociological concepts such as class, race, sexuality, gender, age, and disability
Keuroghlian et al.	2022	United States	Innovation in education; develop new curriculum	Medical students	Design an innovative sexual and gender minority (SGM) curriculum		AOP is not conceptualized The recommendations may align with AOP	Longitudinal SGM health curriculum	Recommendations to incorporate a social determinants of health framework into coursework and weaving SGM health content throughout curriculum
Koch	2021	United States	Theoretical	Nursing students	Provides a perspective on rooting out white supremacy and implementing antiracism	Antiracism contributes to counteracting oppressive structures and beliefs to promote a more just, healthy, and equitable society	AOP is not conceptualized Antiracism overlaps with AOP		Present issues of racism and inequitable access as causes of health disparities; discuss and reflect on topics such as social inequities, systemic racism, White supremacy, and social determinants/drivers of health
Lemieux et al.	2021	United States	Multiple—innovation in education; develop new sessions and empirical; quantitative	MD-PhD students	Describe the impact of a journal club with curricula focused on social justice and anti-racism		AOP is not conceptualized The journal club topics overlap with AOP	Students created an optional intersectional anti-racist journal club to increase awareness of systemic racism	Topics included the history of race and health disparities; provided decision-making power and autonomy to members of the journal club; participants educated the group on a topic and facilitated discussions
Lord et al.	2023	United States	Multiple—innovation in education; develop new course and empirical; quantitative	Medical students	Create a longitudinal, discussion-based antiracism course	Transformative pedagogy facilitates reflection on unexamined personal and institutional assumptions that perpetuate oppression A critical consciousness lens emphasizes authentic liberating dialogue, sharing lived experiences and creating cognitive disequilibrium	AOP is not conceptualized The antiracism course based on transformative pedagogy and critical consciousness focused course overlaps with AOP	Consisted of six 2-h seminars focused on different social systems and four 2-h panel sessions featuring community leaders	Presenting to the larger group to facilitate open-ended discussions; seminars included discussions on race and power, personal and system-level discrimination, and how systems often cause intersectional barriers
Mbaki et al.	2021	UK	Theoretical	Medical students	Propose a toolbox which provides guidance and structure the diversification of curriculum	Decolonisation requires an understanding the history of medical knowledge Proposed framework to create a diverse culturally and ethnically responsive curriculum comprises four categories: initial considerations, historical perspectives, learning environment, and teaching materials and resources	AOP is not conceptualized Incorporation of the framework may overlap with AOP		Reflect on including knowledge from ‘Others’ in the curriculum; clearly explaining how your subject exists in the context of society and discussing the impact of systems on the people who were/are affected; incorporating historical colonial content in curriculum; and providing space for decolonising and diversification discussions
O’Neill & Miller	2015	United States	Innovation in education; curricula changes	Social work students	Describe curricular changes made as a commitment to become an antiracism institution	Antiracism increases critical awareness and analysis of social and structural location in relation to systems of power, privilege, and inequity and implies a pedagogy that fosters a critical consciousness. Antiracism may minimize the effects of other ‘isms’ Antioppressive practices advance attention to diversity, social oppression, and social justice and may decentralize race and racism	AOP is not conceptualized Antiracism overlaps with AOP and suggest AOP should explicitly include race and racism	Two courses consisting of sociocultural concepts and a course about racism	An overview of different forms of oppression; introduce how people are differentially positioned in relation to the dynamics of privilege and oppression; case-based learning; include historical understandings of how racism manifests on micro, mezzo, and macro levels and responding to racism; infuse all courses with an antiracism perspective and
Ona et al.	2020	United States	Multiple—innovation in education; develop new curricula and empirical; qualitative	Medical students	Piloted an antiracism curriculum	An antiracism curriculum entails learning about structural racism, including its historical and continuing manifestations through power, privilege, and policy along with the social epidemiology that links it to health outcomes	AOP is not conceptualized but anti-oppression curriculum needs to occur earlier and more broadly Antiracism overlaps with AOP	A 7.5-h curriculum delivered over 3 sessions	Case studies; didactic material on the history of racism; discussions on implicit bias and health inequities; an exercise in understanding systems of oppression; and a “toolkit for hope”
Power & Sistare	2021	United States	Innovation in education; develop new course and evaluation	Social work students	Pilot a course that explores writing, voice, social justice, and the intersecting issues contributing to incarceration	Anti-oppressive education resists oppression, actively challenges injustice and oppression at the micro level of teaching and the macro level of education reform, and draws together various theoretical traditions, including critical, feminist, queer, and postcolonial perspectives	AOP is conceptualized as resisting and challenging oppression and injustice at the micro and macro level utilizing a variety of theoretical approaches	A 12-week course in which students prepared for upcoming workshops then facilitated writing workshops in the jail and following reflection, returned to the jail and facilitated the modified workshops	Topics covered in readings and classroom discussions included anti-oppressive education, structural oppression in the criminal justice system, ethical issues in jail work, and trauma-informed practice; and reflection on the impact of one’s own power and privilege
Punchhi et al.	2022	Canada	Empirical—qualitative	Medical students and residents	Explore the integration of anti-oppressive pedagogy in medical education through the perspectives of trainees and the reflections of practicing clinicians	Anti-oppressive pedagogy centres oppression and shifts pedagogical approaches towards understanding the drivers of inequities Anti-oppression is the act of challenging inequalities, encompassing principles such as understanding social differences and context, the interaction between personal and political realms and the role of power, and the importance of reflexivity to understand interactions	AOP is conceptualized as understanding the drivers of inequities and centering oppression within the healthcare system		Didactic lectures; online modules; learning through others' lived experiences contextualizes health disparities; discussion and promoting critical thinking; and often includes singular curricular interventions without additional opportunities for dialogue
Robinson et al.	2021	United States	Review	Pharmacy students	Present antiracism teaching as a key modality and an upstream approach to address health disparities	Antiracism teaching is an approach to dismantle health disparities and foster an upstreamist mindset. The upstream concept proposes a shift in addressing health disparities by identifying the root causes of concerns observed “downstream.”	AOP is not conceptualized Antiracism teaching overlaps with AOP	A curriculum was not described. Examples in the literature included a health equity and social justice course with a three-hour session on racism and health equity rounds as a longitudinal activity	Lecture; small-group case discussions; self-reflection activities; incorporate critical race theory; acknowledge race as a social construct and how positions in social hierarchy determine privilege, power, health access and outcomes; and focus on intersectionality
Roy et al.	2022	United States	Innovation in education; develop new course and evaluation	Nursing students	Describe a quality improvement project to integrate the concepts of justice, equity, diversity, and inclusion (JEDI) into the curriculum	A critical race theory (CRT) promotes equity and anti-oppression, posits that systemic racism is a part of society and is embedded within the values, policies, and laws that govern institutions, and seeks to dismantle the manifestations of systemic racism embedded within the health professions curricula	AOP is not explicitly conceptualized but referred to the curriculum as anti-oppressive curricula. This implies that AOP is conceptualized as incorporating the concepts of JEDI and CRT	Curricular changes included adding to a pathophysiology course and workshops with a focus on critical race theory; implicit bias; microaggressions; intersectionality; and White privilege	Case studies; online modules on CRT, social determinants of health, equity, and racial justice; discussions; and group and written reflections
Simaan	2020	UK	Innovation in education; develop new educational activity	Occupation-al therapy	Describe how a study of a Global South community informs transformative education	Decolonisation of a curriculum focuses on non-Western, colonized writers and intellectuals and valorising the knowledges of the colonized	AOP is not conceptualized Decolonising the curriculum overlaps with AOP	A learning activity based on research with olive growers in Palestine to highlight Global South daily lives	Activity involved presenting the author’s study; discussions; watching a film; picking and pickling olives; and writing a reflection about the day
Solomon et al.	2021	United States	Theoretical	Medical students	Advance a framework to dismantle structural racism and show how the framework can be implemented	A 4-step framework for antiracism in medical education based on principles and practices of antiracism consists of: see (become aware of power and advantage), name (teach to name racism), understand (explore consequences of racism with a critical examination), and act (against racism)	AOP is not conceptualized The framework for antiracism in medical education may overlap with AOP		Foster mindfulness and self-awareness; provide implicit bias training with facilitated discussion of racial disparities; and embrace the concepts of the growth mindset and adaptive expertise
Stahl et al.	2022	United States	Multiple—innovation in education; develop new curricula and empirical; mixed methods	Social work students	Designed a capstone course which moved to enacting anti-oppressive approaches	Decision case (DC) analysis explores a broad array of issues and facilitates wrestling with issues of oppression and power, and policy Theatre of the Oppressed (TO) provides tools using theatrical techniques to explore power and undermine oppression	AOP is not explicitly conceptualized but this article refers to teaching an anti-oppression capstone course which implies that the use of DC cases and TO are AOP	A capstone course in the final semester of the curriculum in which students met every other week for eight class sessions	Combined DC and TO approaches; participated in written case analyses and class discussions
Watkins et al.	2018	United States	Innovation in education; curricula descriptions	MA/ PhD psychology students	Explore a decolonial curriculum so knowledges from silenced locations, anti-racist and other decolonial praxes can thrive	Decoloniality defects from imperial and colonial modes of thought and confronts one’s history of privilege Anti-racism/decoloniality curriculum includes acknowledging histories and contexts of inequity; deconstructing marginalization; and integrating philosophies and approaches of non-Western and Indigenous groups	AOP is not conceptualized Anti-racism/decoloniality curriculum overlaps with AOP	Curriculum consists of an array of 40 courses that offer distinctly different alternatives	Teaching about the colonial history; dialogic engagement with theories and critical reflections of daily praxes in communities; introduce liberatory work from “the South”; theater of the oppressed; and anti-racist group work
Wear et al.	2017	United States	Guidance based on the literature AND experience	Medical students	Explore frameworks and practical applications to address racism and other forms of bigotry	Antiracist pedagogy facilitates critical reflection on oppressive power relations and attempts to examine issues from multiple standpoints, so systems of power are made visible Structural competency recognizes the complex ways that matters such as rising income inequalities, decaying infrastructure, poor food distribution networks and other social and economic factors lead to poor health	AOP is not conceptualized Antiracist pedagogy overlaps with AOP	Four-year course on human values in medicine	Emphasizes critical reflection and dialogue; small group setting; discuss issues of power, privilege, identity, and oppression; include short stories, poetry, narrative nonfiction, films, and representations of race and racism; and clinical curriculum experiences that illuminate the impact of structures and elements outside the hospital
Williamson et al.	2022	United States	Multiple—innovation in education; design new modules, theoretical, and provides guidance	Residents	Provide support in learning and teaching for health equity	Equity literacy is the ability to recognize, respond to, redress, cultivate, and sustain efforts to disrupt inequities in various contexts	AOP is not conceptualized. The conceptualization of equity literacy aligns with AOP	Modules that integrate the development of health equity and teaching skills in the curriculum Propose a three-year tiered development model in a residency program with equity literacy outcomes	Identify and pursue self-directed learning about one or two health inequities; include simulation activities and case studies; provide opportunities to serve as members of departmental health equity committees; and work in student-run clinics
